# Intestinal parasitic infections and its association with undernutrition and CD4 T cell levels among HIV/AIDS patients on HAART in Butajira, Ethiopia

**DOI:** 10.1186/s41043-017-0092-2

**Published:** 2017-05-15

**Authors:** Dereje Gedle, Gemechu Kumera, Tewodros Eshete, Kasahun Ketema, Haweni Adugna, Fetuma Feyera

**Affiliations:** 1 Department of Public Health, College of Medicine and Health Sciences, Debre Markos University, Debre Markos, Ethiopia; 2 Department of midwifery, College of Medicine and Health Sciences, Debre Markos University, Debre Markos, Ethiopia; 3 Department of Nursing, College of Medicine and Health Sciences, Debre Markos University, Debre Markos, Ethiopia

**Keywords:** Intestinal parasites, HIV/AIDS, CD4, Undernutrition

## Abstract

**Background:**

Intestinal parasitic infections and HIV/AIDS have been the major public health problems and remain a vital cause of morbidity and mortality in developing countries. Both problems are linked in a vicious cycle. The magnitude of intestinal parasites was prevalent among people living with HIV/AIDS even in the HAART era. However, the pertinent risk factors associated with intestinal parasites among HIV/AIDS patients were not well investigated in Ethiopia particularly at Butajira town. Therefore, the aim of this study was to determine the prevalence of intestinal parasites and associated risk factors among HIV/AIDS patients on HAART in Butajira, Ethiopia.

**Method:**

A cross-sectional study was conducted, and a total of 323 study subjects was involved in the study. A systematic random sampling technique was used to select each participant during data collection. Stool specimen was collected and processed using direct wet mount, formol-ether concentration technique, and modified Ziehl-Neelson staining techniques to identify both common and opportunistic intestinal parasites. Structured questionnaire was used to collect socio-demographic, environmental, clinical, and nutritional data. Both bivariate and multivariate logistic regression analyses were used to assess the association of various explanatory factors on intestinal parasites. *P* value ≤0.05 at 95% CI was considered statistically significant.

**Results:**

The overall prevalence of intestinal parasites was 35.9% (95% CI 31.0–40.9%). Protozoa’s (*Entanmoeba histolytica/dispar* trophozoite, *E. histolytica/dispar* cyst, *Giardia lamblia* trophozoite, *and G. lamblia* cyst), helminths (Tanea species, *Ascaris lumbricoides*, *Strongyloid stercoralis*, Hookworm species *and H. nana*), and opportunistic intestinal parasites (*Cryptosporidium parvum*, *Isospora belli*) were observed in 57 (17.1%), 46 (14.4%), and 28 (8.7%) study participants respectively. Multivariate logistic regression analysis revealed that the presence of animals (AOR 6. 14; 95% CI 3.13, 12.0); using river water (AOR 4.87; 95% CI 1.14, 20.7); undernutrition (AOR 2.59; 95% CI 1.36–4.95); and level of immunosuppression (AOR 4.02; 95% CI 1.78–9.05 and AOR 2.84; 95% CI 1.37–5.89) were significantly associated with intestinal parasites.

**Conclusions:**

The prevalence of intestinal parasites found to be higher among HIV/AIDS patients receiving HAART at Butajira Hospital, southern Ethiopia. Presence of animals, using river water, lower CD4 T cell count, and undernutrition were significant factors affecting intestinal parasites. Therefore, consistent detection of intestinal parasites and deworming of patients should be performed as well as improving health education on personal hygiene, avoiding contact with pit or domestic animals, and using safe or treated water. Furthermore, improving nutritional support and household food access are recommended.

## Introduction

Intestinal parasitic infections have been documented as one of the most significant causes of illnesses and diseases especially among resource limited setting. Globally, over three billion people were infected with one or more intestinal parasites [1]. However, it is a prevalent infectious disease in third world countries where there are socioeconomically poor communities, poor environmental sanitation, overcrowding, and inadequate access to safe water [2, 3]. Intestinal parasites cause high morbidity and mortality of individuals, particularly sub-Saharan countries, and they have been associated with low educational performance, stunting, and physical weakness [4, 5]. Moreover, intestinal parasites are highly related to the spread and progression of human immune deficiency virus/acquired immune deficiency syndrome (HIV/AIDS) [6, 7].

HIV/AIDS and intestinal parasitic infections are linked in a viscous circle [8]. Intestinal parasitic infections, mainly helminths, cause chronic immune activation and twisting the immune response toward T helper-2 immune responses [8–10]. Moreover, they also increase HIV infection and AIDS progression [11–13]. On the other hand, HIV by itself enhances the significance of intestinal parasitic infections; as the progression of AIDS, the risk of opportunistic parasites and diarrhea significantly increased [14–16].

The magnitude of intestinal parasites has been documented higher in different countries of developing countries, particularly in sub-Saharan Africa. Likewise, intestinal parasites are highly distributed in different parts of Ethiopia. Low level of environmental and personal hygiene, unsafe drinking water and food, and improper disposal of human excreta were reported as determinant factors associated with intestinal parasitic infections [17, 18].

There are different reports that showed high magnitude of intestinal parasites among people living with HIV/AIDS (PLWHA) in Ethiopia. Among common intestinal parasites, *E. histolytica*, *G. lamblia*, *A. lumbricoides*, *S. stercoralis* were reported frequently in different studies [19–22]. Similarly, opportunistic parasitic infections such as *C. parvum*, *I. belli*, and *blastocystis* were common feature in HIV/AIDS infections [22, 23].

Intestinal parasitic infection is one of the major causes of morbidity, and it is highly prevalent among PLWHA in different parts of Ethiopia. However, the burden of both common and opportunistic intestinal parasites among HIV/AIDS patients receiving HAART is not well studied in the study area. Furthermore, determinant factors affecting intestinal parasites, especially malnutrition and cluster differentiation (CD4) T cell count were not comprehensively assessed in relation to intestinal parasites. Therefore, the aim of this study was to determine the prevalence of intestinal parasites and associated risk factors among HIV/AIDS patients on HAART in Butajira, Ethiopia.

## Methodology

### Materials and method

#### Study design, period, and area

An institution-based cross-sectional study was conducted at Butajira Hospital, Gurage Zone, Southern Nations, Nationalities, and People’s Region (SNNPR), Ethiopia, between September 2015 and May 2016. Butajira hospital is found at Butajira town, which is a generalized hospital with 110 beds that gives health care for resident of Butajira and surrounding area. The town lies in the average at 2100 m above sea level and somewhat has warm climate. The hospital catchment area populations are estimated around 1.3 million. A total of 1110 HIV/AIDS patients have been registered to ART care clinic at Butajira Hospital, of which 450 were in pre-ART care, and 660 were receiving HAART.

##### Operational definition

*Undernutrition*: the study participants whose body mass index less than 18.50 kg/m^2^ based on WHO classification for malnutrition using body mass index [24].

*Household food insecurity access scale* is a measure of household food insecurity of study subjects in the past 4 weeks. It was calculated based on Food and Nutrition Technical Assistance standardized tools. Then, it was classified into Food Secure, Mildly Food Insecure Access, Moderately Food Insecure Access, and Severely Food Insecure Access [25].

*Household dietary diversity* is the patient’s economic ability to access a diversity of foods during the past 1 day (24 h.). Twelve of the questions were used to assess dietary diversity based on Food and Agriculture Organization standardized tools. Then, the mean dietary diversity score in the study participants was computed. Finally, the tertiles of the dietary diversity score were calculated with the highest tertile categorized as adequate food diversity, while the lowest tertiles were inadequate food diversity [26].

*Anemia status* was classified by using the level of hemoglobin concentration in grams per deciliter based on WHO classifications. Hemoglobin level was ranged between 12 and 16 g/dl, and 13–17 g/dl was considered as normal for female and male patients respectively. The study participants were classified as anemic for male and female when the hemoglobin concentration was <13 and <12 g/dl respectively [27].

*Drug adherence status* was estimated by percent of missed dose for the last 6 months. It was extracted from patients’ chart combined with self-reported adherence measurement technique. Then classified based on WHO classifications into 1 = Good adherence: if the average adherence is greater than 95% (patients missed <2 doses of 30 doses or <3 doses of 60 doses); 2 = Fair adherence: if the average adherence is 85–94% (patients missed 3–5 doses of 30 doses or 3–9 doses of 60 doses); 3 = Poor adherence: if the average adherence is <85% (patients missed >6 doses from 30 doses or >9 doses of 60 doses) [28].

*Level of immunosuppression* was determined by CD4 T cells level based on WHO classifications. Then, categorized into severe, moderate, mild, and not significant immunosuppression if patients’ CD4 T cells level were <200, 200–349, 350–500, and >500/mm^3^ respectively [29].

#### Sample size and sampling technique

The sample size was determined using single population proportion formula taking prevalence of intestinal parasites among PLWHA receiving HAART in Addis Ababa, Ethiopia, (27.8%) [30] with 5% marginal error and 95% confidence interval (CI) of certainty (alpha = 0.05). In this study, 5% of the non-response rate was taken, and the final sample size was 323. Therefore, 323 study participants were included in the study.

A systematic random sampling technique was used to select the study subjects. A systematic random sampling technique was used considering Butajira hospital ART clinic gave the service on the average 15 patients per day, and 660 patients were expected to visit the hospital in 3 months of data collection period. Since the sample size was 323, the sampling interval was determined two. From the first two subjects, one patient was randomly selected by lottery method, so every other patient was selected to participate in the study.

#### Study populations and data collection

The study populations were all adult PLWHA who were currently receiving HAART. Patients who were receiving HAART and involved in ART follow up clinic at Butajira hospital and aged 18 years and above were included in the study.

The data was collected from April to June 2016 using a structured questionnaire. Socio-demographic characteristics, nutrition, clinical-related characteristics, and laboratory data were collected. Two clinical nurses and two laboratory technicians were recruited and 2 days training was given. The data collection process was followed daily by the principal investigator. Data on opportunistic disease in the past 6 months, WHO clinical stages of disease, drug adherence and chronic diseases were obtained from patient charts. The CD4+ T cell count was measured by BD FACS calibur machine (US) and categorized according to its clinical significance. Hemoglobin was measured with CELL-DYN hematology analyzer (US).

#### Stool sample collection and processing

Appropriate amount of fresh stool sample was taken and processed at parasitology laboratory, Butajira hospital using standard procedure. In the case of formol-ether concentration method, about 1 g of stool sample was mixed with 7 ml formalin saline in a clear 15-ml conical centrifuge tube. Suspension was filtered through a sieve into second conical tube. After pouring 3 ml of diethyl ether into the content and centrifuged for 5 min at medium speed, approximately 2500 rpm. The supernatant was discarded by tilting the tube and pour off all the fluid. The sediment mixed with the remaining small fluid and about two drops of the deposit was placed on a slide, to which a drop of iodine solution was added and covered with cover slide. The supernatant was poured away and the tube was replaced in its track. Finally, smear was prepared from the sediment and observed under light microscope with a magnification of ×100 and ×400[31]. In the case of modified Ziehl-Neelsen staining method, a portion of stool sample was processed for detection of *C. parvum and I. belli*. Briefly, thin smear was prepared directly from sediment of concentrated stool then allowed to air dry and flamed for a few seconds for sterilization. The slide was exposed with methanol for 5 min and stained with carbol fuchsine for 30 min. The slide was washed with tap water and decolorized with 5% sulfuric acid for 1–3 min. The slide was then rinsed in tap water and observed under light microscope with a magnification of ×1000 [31].

#### Quality control

The questionnaire was adapted and modified in our context from previous literatures. It was first prepared in English and then translated into the local language, Amharic, and then retranslated back to English by an expert to maintain its consistency. Training was given for data collectors and supervisor. Pre-testing of the questionnaire was conducted on 20 patients receiving ART in the nearby health center 3 weeks prior to the actual survey. The data collection process was strictly followed day to day by the supervisor and principal investigator. Aseptic techniques and standard operating procedures were followed during sample collection, processing, transportation, and identification of intestinal parasites and measuring blood samples.

#### Data analysis and interpretation

The data was checked for completeness, coded, and first entered into EPI-info version 7, then it was rechecked and transferred to the Statistical Package for Social Science (SPSS) version 20 for analysis. Chi-square was used to carry out a descriptive analysis. To assess the effect of the various factors related to intestinal parasites, bivariate and multivariate logistic regression analyses were used. The absence of multi-co-linearity was checked by using VIF/tolerance. The model adequacy was checked by using Hosmer and Lemeshow goodness of fit test. *P* value ≤0.05 at 95% CI was considered statistically significant.

## Results

### Socio-demographic characteristics of the study participants

A total of 323 HIV infected participants on HAART were included in this study. The age of participants ranges from 22 to 78 years with a median of 39 years. More than three fifth of participants (61.5%) were in the age group of 30–44 years and 26.9% were above 44 years. The majority of the study participants (63.2%) were women, and 50.8% of participants were married. Most of the participants (86.4%) had low monthly income and 58.5% were urban dweller. Sixty-two (19.2%) participants were daily laborers as shown in (Table 1).

**Table 1:** Socio-demographic characteristics of the study subjects at Butajira Hospital, southern Ethiopia, 2014, (*n* = 323)

Characteristics	Frequency (*n*)	Percent (%)
Sex		
Male	119	36.8
Female	204	63.2
Age (years)		
18–29	37	11.5
30–44	199	61.5
≥ 45	87	26.9
Residence		
Urban	189	58.5
Rural	134	41.5
Marital status		
Single	19	5.9
Married	164	50.8
Divorced	44	13.6
Widowed	86	26.6
Separated	10	3.1
Educational status		
Can read and write	129	39.9
Cannot read and write	39	12.1
Primary education	86	26.6
Secondary education	49	15.2
Tertiary education	20	6.2
Religion		
Orthodox	153	47.4
Muslim	106	32.8
Protestant	62	19.2
Catholic	2	0.6
Ethnicity		
Gurage	192	59.4
Silitie	56	17.3
Amhara	43	13.3
Oromo	14	4.3
Hadiya	17	5.3
Occupation		
Governmental employer	41	12.7
Self-employer	55	17.0
Farmer	42	13.0
Merchant	50	15.5
Daily laborer	62	19.2
House wife	51	15.8
Jobless	22	6.8
Monthly income (ETB)		
<1000	279	86.4
≥1000	44	13.6
Family size		
≤3	156	48.3
4–6	146	45.2
>6	21	6.5

### Environmental, clinical, and nutritional profiles of the study participants

Most of the study participants (95.7%) have a toilet in site their home, and 23.2% participants had domestic (pit) animals. Twenty two (6.8%) participants used river water as a source of water for basic activities. Fifty-four (16.7%) of individuals had one or more gastrointestinal symptoms, of which diarrhea (8.7%) was the highest rank followed by mal-digestion (8.0%). The median CD4+ T cell count and hemoglobin concentration level of participants were 403 cells/μl with 326 inter quartile range (IQR) and 13.1 g/dl with 6 IQR respectively. More than three fifth of participants were at WHO clinical stage one, and 36.2% of participants have their CD4 T cells count greater than 500 cells/μl. Eighty-eight (27.2%) participants were anemic; however, most of them (91%) were with good drug adherence. About 55.4% of participants were initiated with HAART care more than 3 years prior to data collection period (Table 2).

**Table 2:** Environmental, clinical, and nutritional profiles of the study participants at Butajira Hospital, southern Ethiopia, 2014, (*n* = 323)

Variables	Frequency (*n*)	Percent (%)
Presence of animals		
No	248	76.8
Yes	75	23.2
Presence of toilet		
No	14	4.3
Yes	309	95.7
Source of water		
Tape	301	93.2
River	22	6.8
Eating difficulty		
No	244	75.5
Yes	79	24.5
Loss of appetite	70	21.7
Vomiting	23	7.1
Nausea	14	4.3
Swallowing difficulty	6	1.9
Gastrointestinal symptoms		
No	269	83.3
Yes	54	16.7
Diarrhea	28	8.7
Indigestion	26	8.0
Constipation	8	2.5
WHO clinical stage		
Stage I	196	60.7
Stage II	58	18.0
Stage III	62	19.2
Stage IV	7	2.2
CD4+ T cell count (cells/μl)		
<200	53	16.4
200–349	74	22.9
350–500	79	24.5
>500	117	36.2
Anemia status		
Normal	235	72.8
Anemic	88	27.2
Current/past OI in the past 6 months		
No	189	58.5
Yes	134	41.5
Duration of HAART		
<6 months	40	12.4
6–12 months	48	14.9
1–3 years	56	17.3
>3 years	179	55.4
Drug adherence		
Good	294	91.0
Fair	21	6.5
Poor	8	2.5
Nutritional status		
Severe malnourished	10	3.1
Moderate malnourished	21	6.5
Mild malnourished	51	15.8
Nourished	201	62.2
Over weight and obese	40	12.4
Household food insecurity status		
Food secured	68	21.1
Mildly food in secured	16	5.0
Moderately food in secured	104	32.2
Severely food in secured	135	41.8
Household dietary diversity		
Inadequate	128	39.6
Adequate	195	60.4

Regarding nutritional status of the study participants, the overall prevalence of undernutrition among PLWHA on HAART was 25.4%. Mild, moderate, and severe malnutrition was observed in 15.8, 19 6.5, and 3.1% participants respectively. The majority of participants (78.8%) were food insecure, of which 5.0, 32.2, and 41.8% were mildly, moderately, and severely food insecure respectively. Moreover, about 128 (39.7%) individuals were with inadequate dietary diversity (Table 2).

### Prevalence of intestinal parasites among the study participants

The overall prevalence of intestinal parasites among HIV/AIDS patients receiving HAART was 35.9% (95% CI 31.0–40.9%). Protozoas, helminths, and opportunistic intestinal parasites were observed in 57 (17.1%), 46 (14.4%), and 28 (8.7%) participants respectively. The most prevalent protozoan parasites, helminths, and opportunistic intestinal parasites among HIV/AIDS patients were *E. histolytica/dispar* trophozoite (7.1%), Tanea species (7.4%), and *C. parvum* (5.9%) respectively. Infected participants were also found to harbor multiple types of parasites. Multiple parasitic infections were observed in 1.5% of the total examined patients (Table 3).

**Table 3:** Intestinal parasites co-infection among the study participants at Butajira Hospital, southern Ethiopia, 2014, (*n* = 323)

Intestinal parasites	Frequency (*n*)	Percent (%)
Negative	207	64.1
Positive	116	35.9
Protozoas		
*E. histolytica/dispar* trophozoite	23	7.1
*E. histolytica/dispar* cyst	15	4.6
*G. lamblia* trophozoite	17	4.8
*G. lamblia* cyst	2	0.6
Helminthes		
Tanea species	24	7.4
*A. lumbricoides*	10	3.1
*S. stercoralis*	5	1.5
Hookworm species	2	0.7
*H. nana*	5	1.5
Opportunistic parasites		
*C. parvum*	19	5.9
*I. belli*	9	2.8
Multiple parasitic infections		
*E. histolytica/dispar* trophozoite *+ G. lamblia* trophozoite	2	0.6
*E. histolytica/dispar* trophozoite *+ A. lumbricoides*	2	0.6
*G. lamblia trophozoite +* Tanea species	1	0.3

### Intestinal parasites and possible risk factors

All the study variables computed by bivariate analysis were also investigated using multivariate analysis. In multivariate stepwise logistic regression analysis, out of nine selected variables in bivariate analyses, only four variables (presence of animals, source of water, CD4 T cell count, and undernutrition) were retained and significantly associated with intestinal parasites. The remaining five variables (age, educational status, income, presence of toilet, WHO clinical stages) were failed to associate significantly with intestinal parasites in the multivariate model (Table 4).

**Table 4:** Risk factors associated with intestinal parasites among PLWHA receiving HAART at Butajira Hospital, southern Ethiopia, 2014, (*n* = 323)

Predictors	Intestinal parasites		COR (95% CI)	AOR (95% CI)	*P* values
	Yes	No			
Residence					
Urban	62	127	1		
Rural	80	54	1.38 (0.87–2.19)		
Educational status					
Unable to read and write	55	74	4.21 (1.17–15.0)		
Able to read and write	14	25	3.17 (0.79–12.7)		
Primary education	23	63	2.06 (0.55–7.72)		
Secondary education	28	21	4.25 (1.10–16.4)		
Tertiary education	3	17	1		
Income (ETB)					
<1000	107	172	2.42 (1.12–5.23)		
≥1000	9	35	1		
Presence of animal					
Yes	51	24	5.98 (3.41–10.5)	6.14 (3.13–12.0)	0.00**
No	65	183	1	1	
Presence of toilet					
Yes	103	206	1		
No	13	1	26 (3.3–201)		
Source of water					
Tape	98	203	1	1	
River	18	4	9.32 (3.07–28.3)	4.87 (1.14–20.7)	0.03*
WHO stages					
Stage I	66	130	1		
Stage II	30	28	2.11 (1.16–3.82)		
Stage III	18	44	0.81 (0.43–1.50)		
Stage IV	2	5	0.78 (0.15–4.17)		
CD4 T cell count					0.00**
<200	31	22	3.74 (1.89–7.39)	4.02 (1.78–9.05)	
200–349	33	41	2.13 (1.16–3.94)	2.84 (1.37–5.89)	
350–500	20	59	0.90 (0.47–1.72)	1.01 (0.46–2.20)	
>500	32	85	1	1	
Undernutrition					
Yes	44	38	2.72 (1.62–4.54)	2.59 (1.36–4.95)	0.004*
No	72	169	1	1	

Stepwise (Backward LR) was used in logistic regression. Hosmer and Lemeshow test was at *P* = 0.31

N.B α = 0.05

*Significant, **Highly significant

In this study, the presence of one or more animals in the house were significantly associated with intestinal parasites (*P* = 0.00). Participants who had one or more animals in their house were six times more likely to be infected with one or more intestinal parasites as compared with those free animals in their home (AOR 6.14; 95% CI 3.13, 12.0). Using river water was also significantly associated with intestinal parasites (*P* = 0.03). Participants whose source of drinking water were river water were four times more likely to be infected with one or more intestinal parasites as compared with those using tap water (AOR 4.87; 95% CI 1.14, 20.7). Intriguingly, CD4 T cell lymphocyte was highly associated with intestinal parasites (*P* = 0.00). Immunocompromised patients whose CD4 T cell lymphocytes less than 200 and 200–349 cells/μl were more likely to be infected with one or more intestinal parasites as compared to those who had above 500 cells/μl, (AOR 4.02; 95% CI 1.78–9.05) and (AOR 2.84; 95% CI 1.37–5.89) respectively. Moreover, undernutrition were significantly associated with intestinal parasites (*P* = 0.004). Under-nourished patients were 2.59 times more likely to be infected in one or more intestinal parasites as compared with nourished individuals (AOR 2.59; 95% CI 1.36–4.95).

## Discussion

This study revealed the prevalence of intestinal parasites and associated risk factors among HIV/AIDS patients receiving HAART at Butajira Generalized Hospital. The overall prevalence (35.9%) of intestinal parasites among PLWHA receiving HAART remains significant. This finding was relatively higher than previous studies conducted in France (22%), Brazil (14%), Nepal (22.4%), Congo (8.5%), Cameron (24.6%), and Nigeria (15.3%) [32–37]. However, the result of this study was lower than a study conducted in Iran (67.7%) and in different countries of Ethiopia including in selected ART clinic Adama, Afar, Dire-Dawa (48%), and Bahirdar (69%) [22, 38, 39]. On the other hand, Telele et al. and Missaye et al. also reported 24.3 and 17.3% prevalence of intestinal parasites in Gondar and Dessie, Ethiopia, respectively [22, 40]. The discrepancy of the prevalence among different parts of the country may reflect the existence of diverse ethnic experiences and implementing prevention and treatment measures against intestinal parasites. Furthermore, it is wise to expect the existence of variation in geographic location and general hygiene of the population.

In the current study, we found that the presence of one or more animals in the house was significantly associated with intestinal parasites (*P* = 0.00). Participants who had one or more animals in their house were six times more likely to be infected with one or more intestinal parasites as compared to those who had no animals. This finding was in accordance with Mohan et al. [41] and Dwivedi et al. [42]. The association might be due to living of human with domestic animals which increases a tendency to contact with pit or domestic animal excretion and consuming their products. So, those patients will be more vulnerable to be infected with one or more intestinal parasites [43, 44]. *C. parvum* and *giardia* are ubiquitous parasites in mammals, particularly domestic animals and those protozoan were commonly identified in dogs, sheep, and horses [45–47].

Participants whose source of water were river water were four times more likely to be infected with one or more intestinal parasites as compared to those using tap water (*P* = 0.03). Similar results were found in studies conducted in Nigeria, Malaysia, and Ethiopia [20, 48, 49]. In fact, river/unprotected water is highly contaminated with animals and human excretion since people are usually bathing and washing their clothes under river. These habits have been practiced in developing countries particularly in Ethiopia due to scarcity or inadequate distribution of safe/clean water. So, using untreated/unsafe water contributes to be infected with one or more intestinal parasitic infections [50, 51]. The most prevalent waterborne intestinal parasites producing diarrhea are *cryptosporidiosis*, *giardiasis*, and *E. histolytica*. These parasitic infections have been commonly reported in immunocompromised patients, particularly in HIV/AIDS patients [51].

The other pertinent finding was CD4 T cell count which was highly associated with intestinal parasites (*P* = 0.00). Immunocompromised patients whose CD4 T cells below 350 cells/μl were more likely to be infected with a particular intestinal parasite. This is in agreement with previous findings in Nigeria [37], Nepal [34], and different parts of Ethiopia like Addis Ababa [30], Hawasa [16], and Dessie [20]. The reduction in CD4 T cell count in PLWHA predisposes to intestinal parasites, especially opportunistic intestinal parasitic infections [52–54]. Immuno-deficient patients are more susceptible to acquiring intestinal parasites, and they are unable to clear once infection is established [55].

Moreover, undernutrition was significantly associated with intestinal parasites (*P* = 0.004). Participants who were under-nourished 2.59 times more likely to be infected with one or more intestinal parasites as compared to nourished individual. Infectious diseases and malnutrition have interconnected one another in a viscous cycle. Malnourished individuals are more vulnerable to be infected with a particular intestinal parasite as compared to nourished individuals because malnourished individuals lead to decreased immune function, mucosal damage, and increased invasion by intestinal parasites. Furthermore, malabsorption, loss of appetite, and diarrhea, all of which, lead to nutrient losses and further damage to defense mechanisms [56, 57].

The limitation of the current study could be other laboratory technique like water-ether sedimentation method which was not used for detection of microsporidia. In addition, we have not used molecular techniques and immune-fluorescent techniques sensitive for parasites. Besides, assessment of household dietary diversity and household food insecurity status depends on the past 24-h and 1-month period recall method, respectively, which might create a possibility of recall bias.

## Conclusions

The prevalence of intestinal parasites found to be higher among HIV/AIDS patients receiving HAART at Butajira Hospital, southern Ethiopia. Presence of animals, using river water, lower CD4 T Cell count, and undernutrition were significant factors affecting intestinal parasites. Routine and consistent detection of intestinal parasites should be performed. Moreover, improving public health measures that should emphasize on personal hygiene, avoiding contact with pit or domestic animals, using safe or treated water, and improving nutritional support and household food access are recommended.

## Abbreviations

AIDS: Acquired immunodeficiency syndrome
AOR: Adjusted odds ratio
BD FACS: Becton Dickson fluorescent activated cell sorter
CD: Cluster of differentiation
HAART: Highly active anti-retroviral therapy
HIV: Human immunodeficiency virus
PLWHA: People living with HIV/AIDS
SNNPR: Southern Nations, Nationalities, and People’s Region
SPSS: Statistical package for social science
WHO: World Health Organization
